# Associations between Urinary Advanced Glycation End Products and Cardiometabolic Parameters in Metabolically Healthy Obese Women

**DOI:** 10.3390/jcm8071008

**Published:** 2019-07-10

**Authors:** Estifanos Baye, Alicja B Mark, Malene W Poulsen, Jeanette M Andersen, Lars O Dragsted, Sussane G Bügel, Barbora de Courten

**Affiliations:** 1 Monash Centre for Health Research and Implementation, School of Public Health and Preventive Medicine, Monash University, Melbourne, VIC 3168, Australia; 2 Department of Nutrition, Exercise and Sports, University of Copenhagen, 1165 København, Denmark

**Keywords:** advanced glycation end products, carboxymethyl-lysine, methylglyoxal-derived hydroimidazolone, insulin resistance, type 2 diabetes, cardiovascular disease

## Abstract

Advanced glycation end products (AGEs) have been implicated in the pathophysiology of type 2 diabetes and cardiovascular disease. We aimed to determine the associations of urinary carboxymethyl-lysine (CML) and methylglyoxal-hydroimidazolone (MG-H1) levels with cardiometabolic parameters in metabolically healthy obese women. Anthropometric, glycemic, cardiovascular, and urinary AGE parameters were measured in 58 metabolically healthy obese women (age: 39.98 ± 8.72 years; body mass index (BMI): 32.29 ± 4.05 kg/m^2^). Urinary CML levels were positively associated with BMI (*r* = 0.29, *p* = 0.02). After adjustment for age and BMI, there was a trend for positive associations between urinary CML levels and fasting (*p* = 0.06) and 2 h insulin (*p* = 0.05) levels, and insulin resistance measured by homeostatic model assessment (HOMA-IR) (*p* = 0.06). Urinary MG-H1 levels were positively associated with systolic and diastolic blood pressure, pulse pressure, mean arterial pressure, and total and low-density lipoprotein cholesterol after adjustment for age, BMI, and HOMA-IR (all *p* ˂ 0.05). There were no associations between urinary CML levels and cardiovascular parameters, and between urinary MG-H1 levels and glycemic measurements. Our data support a role of urinary AGEs in the pathophysiology of insulin resistance and cardiovascular disease; however, future studies are highly warranted.

## 1. Introduction

Advanced glycation end products (AGEs) are formed when proteins or lipids become non-enzymatically glycated after exposure to sugars [1]. AGEs are formed endogenously at lower rates under normal physiological conditions [2], but their formation is increased in patients with impaired glucose metabolism [3]. Accumulation of AGEs has been implicated in the development of several chronic diseases, including type 2 diabetes (T2DM), cardiovascular disease (CVD), and neurodegenerative disease (Alzheimer’s and Parkinson’s diseases) through altering the structure and functions of proteins or by increasing inflammation and oxidative stress [4]. 

Carboxymethyl-lysine (CML) and methylglyoxal-derived hydroimidazolone (MG-H1) are the most commonly measured and well-described non-fluorescent AGEs in blood, urine, and feces [4]. CML and MG-H1, which are derived from lysine and arginine, are important to estimate overall AGE exposure [5]. The measurement of protein bound AGEs requires complicated sample preparation or the development of stable antibodies [6]. Urinary AGEs, which is easily accessible in a non-invasive manner and can be detected using relatively easily and inexpensive methods, have been suggested to reflect circulating AGEs in healthy individuals with normal renal function [6,7]. Published studies, however, have shown contradictory findings with regards to the relationships between urinary AGE levels and cardiometabolic risk factors. One study reported positive associations between urinary AGE levels and anthropometric and metabolic parameters in healthy individuals and patients with metabolic syndrome [6], while other studies in individuals with and without T2DM did not [7,8]. However, these studies reported either total urinary AGEs or CML levels [6,7,8], and no studies have thus far investigated the associations between urinary MG-H1 levels and cardiometabolic parameters. The hypothesis of the present study was to determine whether urinary CML and MG-H1 levels were associated with cardiometabolic parameters in metabolically healthy obese women. 

## 2. Materials and Methods 

### 2.1. Study Design and Population 

This was a cross-sectional analysis of baseline data from a randomized controlled trial that was conducted in healthy overweight and obese Caucasian women at the Department of Nutrition, Exercise, and Sports, University of Copenhagen, Denmark. As previously reported [5], women aged 20–50 years with a body mass index (BMI) of 25–40 kg/m^2^ and a waist circumference of >88 cm were included in the study. Participants were excluded from the study if they were current smokers, pregnant/lactating, vegetarian/vegan, physically active for more than 8 h per week, or allergic to para-aminobenzoic acid. Women who had weight change of >3 kg in the previous two months, known medical or postmenstrual conditions, gastric bypass surgery, or those who had donated blood in the last three months or used supplements/medications were also excluded. The study population was well characterized with regard to anthropometric, metabolic, cardiovascular, and AGEs measurements. Participants were asked to restrain from rigorous physical activity and alcohol consumption for 48 h before all measurements. This study was carried out in accordance with the recommendations of local ethics guidelines, Danish Research Ethics Committee. The protocol was approved by the Danish Research Ethics Committee (#H-4-2011-077). All subjects gave written informed consent in accordance with the Declaration of Helsinki.

### 2.2. Anthropometric Measurements 

Weight, height, and waist and hip circumferences were measured using standard equipment and methods. BMI was calculated as weight (kg)/height (m) square, while waist-to-hip ratio (WHR) was computed as waist (cm)/hip (cm). 

### 2.3. Cardiovascular Measurements 

Blood pressure was measured using an automated oscillometric measurement and was assessed in a sitting position after 30 min of rest. The mean of three measurements, separated by 5 min, was recorded. Pulse pressure was calculated by subtracting diastolic blood pressure from systolic blood pressure. Mean arterial pressure was computed using ((diastolic blood pressure × 2) + systolic blood pressure)/3. Plasma lipid levels including total cholesterol, triglycerides, low-density lipoprotein (LDL), and high-density lipoprotein (HDL) cholesterol were measured using an enzymatic colorimetric method (Horiba, Montpellier, France). 

### 2.4. Metabolic Measurements

A 75 g oral glucose tolerance test (OGTT) was performed after a 12 h overnight fast and blood samples were drawn at 0 and 120 min to determine the participant’s glucose tolerance status. Plasma glucose was measured using an enzymatic calorimetric method (ABX Pentra, Glucose HK CP, Montpellier, France), while insulin in serum was determined by a chemiluminescent immunometric assay (Siemens healthcare Diagnostics, West Sacramento, CA, USA). Fasting insulin levels were not available from two participants. Insulin resistance measured by homeostatic model of assessment (HOMA-IR) was computed as (glucose (mmol/l) × insulin (µU/mL))/22.5. Insulin secretion determined by homeostatic model of assessment (HOMA-B) was calculated using the formula: (insulin (µU/mL) × 20)/(glucose (mmol/L) − 3.5) [9]. Insulin sensitivity index (ISI_0, 120_) was calculated using fasting and 2 h glucose and insulin values obtained from the OGTT, as described elsewhere [10]. 

### 2.5. Measurement of AGE Levels

Twenty-four hour urine samples were collected and kept cold in thermo bags throughout the collection period, and then stored at −80 °C immediately after return of the samples until analysis. An ultra-performance liquid chromatography-triple quadruple detector system (Waters, Milford, MA, USA) was used to determine urinary CML and MG-H1 levels, as described previously [5,11]. Briefly, an Oasis HLB LP 96-well plate (60 mg; Waters, Hedehusene, Denmark) was used to pre-concentrate the samples by solid phase extraction (SPE). The SPE cartridges were preconditioned with 1 mL methanol followed by two washes with the same volume of water. A total of 100 µL urine together with 10 µL internal standards (30 µg/mL; PolyPeptide Group, Torrance, CA, USA) was loaded onto the SPE cartridge and eluted with 300 µL 20% methanol/water. The loading and the eluate were combined, and the solvent was evaporated. The samples were then re-dissolved in 200 µL 26 mmol/L ammonium formate. Liquid chromatography tandem mass spectrometry operated in the multiple-reaction mode was used to analyze the samples on a 2.1 mm × 15 cm hypercarb column (3 μm particle size; Thermo Fischer Scientific, Waltham, MA, USA). The gradient used was 0–20% acetonitrile/26 mmol/L ammonium formate in 0–3 min and 20–60% in 3–10.2 min, and then immediately back to 100% 26 mmol/L ammonium formate to recalibrate the column for 3.8 min before the next injection. The flow rate was 0.1 mL/min, and the transitions used for quantification of MG-H1 and CML were 229 > 166 and 205 > 130, respectively. A Millipore ultra-pure water system was used to purify the water used for all solutions. Calibration curves were prepared for CML and MG-H1 using six concentration levels (0, 0.16, 0.31, 0.63, 1.25, and 2.50 μg/mL). The linearity in this concentration range was 0.9995 for CML, and 0.9998 for MG-H1 levels.

### 2.6. Statistical Analyses

Normality of distributions was assessed, and skewed parameters were logarithmically transformed to approximate to normal distribution. Means and standard deviations (SD) were reported unless otherwise stated. Pearson correlation tests were performed to examine the correlations between levels of urinary AGEs and cardiometabolic parameters. The associations between urinary AGEs levels and cardiometabolic parameters were determined using multiple linear regression analyses after adjustment for age, BMI or WHR, and HOMA-IR. Analyses were performed using SAS Studio 3.4 (SAS Institute Inc, Cary, NC, USA) and *p*-values were reported to be significant at *p* < 0.05.

## 3. Results

### 3.1. Participant Characteristics

Fifty-eight metabolically healthy women aged 39.98 ± 8.72 years with a BMI of 32.39 ± 4.05 kg/m^2^ and WHR of 0.92 ± 0.05 were included in the analysis. The average systolic and diastolic blood pressure was 123.28 ± 13.91 mmHg and 82.26 ± 9.62 mmHg, respectively. Participants had a mean total cholesterol of 5.04 ± 0.72 mmol/L, LDL of 3.10 ± 0.68 mmol/L, and HDL of 1.37 ± 0.28 mmol/L. The mean fasting and 2 h glucose levels of the participants were 5.33 ± 0.31 and 5.75 ± 1.09 mmol/L, respectively (Table 1). 

### 3.2. Correlation Analyses

The results of the correlation analyses between AGE levels and cardiovascular and metabolic parameters are presented in Table 2. 

Urinary CML levels were positively correlated with BMI (*r* = 0.29, *p* = 0.02), fasting insulin (*r* = 0.37, *p* = 0.004), 2 h insulin (*r* = 0.36, *p* = 0.01), HOMA-IR (*r* = 0.37, *p* = 0.004), and HOMA-B (*r* = 0.36, *p* = 0.01). There was also a trend for a negative relationship between urinary CML and ISI_0, 120_ (*r* = −0.22, *p* = 0.05). Cardiovascular parameters including blood pressure and lipid levels did not correlate with urinary CML levels (Table 2). 

Urinary MG-H1 levels were correlated with systolic blood pressure (*r* = 0.33, *p* = 0.01), diastolic blood pressure (*r* = 0.26, *p* = 0.04), pulse pressure (*r* = 0.27, *p* = 0.04), mean arterial blood pressure (*r* = 0.3, *p* = 0.02), and total cholesterol (*r* = 0.26, *p* = 0.04), but not with anthropometric and glycemic parameters (all *p* > 0.09). 

### 3.3. Multiple Linear Regression Analyses

Results of the regression analyses are presented in Table 3. After adjustment for age and BMI, there was a trend for positive associations between urinary CML levels and fasting (*p* = 0.06), 2-h insulin (*p* = 0.05) and HOMA-IR (*p* = 0.06). Urinary MG-H1 levels were positively associated with systolic (*p* = 0.01) and diastolic (*p* = 0.01) blood pressure, pulse pressure (*p* = 0.02), mean arterial blood pressure (*p* = 0.01), total cholesterol (*p* = 0.01) and LDL cholesterol (*p* = 0.05) after adjustment for age, BMI and HOMA-IR. The associations between urinary CML levels and cardiovascular parameters, and between urinary MG-H1 levels and anthropometric and glycemic measurements remained non-significant after adjusting for covariates. In addition, urinary CML was not associated with ISI_0, 120_ after adjusting for age and BMI. In all multiple linear regression analyses, results were unchanged by replacing BMI with WHR. 

## 4. Discussion

We investigated the associations between urinary AGE levels and cardiometabolic parameters in obese, but metabolically healthy Caucasian women. We found that urinary CML levels were positively associated with obesity and markers of insulin resistance such as fasting and 2 h insulin levels and HOMA-IR, while urinary MG-H1 levels were associated with cardiovascular parameters including systolic and diastolic blood pressure, pulse pressure, mean arterial pressure, and total and LDL cholesterol. 

We report a positive association between urinary CML levels and obesity measured by BMI. Other studies that involved non-diabetic individuals, however, demonstrated that urinary AGE levels were not associated with BMI [7,8]. These studies measured small-sized AGE peptides by flow injection assays, which do not identify any particular AGE. Moreover, a study by Maza and colleagues [7] involved only adult men, while the other study included elderly subjects aged 70 to 89 years [8], which could have potentially influenced the association. Obesity is known to increase oxidative stress [12], and CML levels, which are formed from the oxidative degradation of fructosyl-lysine and by the reactions of glyoxal and ascorbic acid with lysine residues in proteins [13,14], have been shown to associate with oxidative stress [13]. Our finding supports the important link between urinary CML levels and the development of obesity. 

We demonstrated that urinary CML levels were associated with markers of insulin resistance such as fasting and 2 h insulin levels and HOMA-IR, but not with cardiovascular parameters, after adjustment for age and BMI or WHR. Other studies have also found that urinary CML levels were not associated with fasting glucose levels [6,7,8]. A study in elderly people showed no association between urinary AGEs and insulin levels in both individuals with and without T2DM [8]. The discrepancy could be the result of a difference in the age of the participants or in the type of AGEs assessed in urine. We determined specific urinary AGEs (CML and MG-H1 levels) in adult women, while the other study [8] measured total AGE levels in an elderly population. CML has been shown to activate inflammatory signaling pathways that contribute to the development of insulin resistance in obese individuals [15]. Related to this, urinary CML levels have been reported to be elevated in individuals with T2DM compared with those without T2DM [16,17]. Our data suggest that urinary CML plays a role in the pathophysiology of insulin resistance in obese individuals even before hyperglycaemia is evident; however, future studies are required to confirm this. 

Methylglyoxal, a highly reactive carbonyl species, is formed by lipid peroxidation, degradation of glycolic intermediates, and glycated proteins [18]. It has been shown to inhibit antioxidant enzymes through binding of free sulfhydryl groups at their active sites, and thereby cause oxidative stress [19]. MG-H1 levels have been suggested to play an important role in the development of carbonyl stress-induced conditions including hypertension, dyslipidaemia, and atherosclerosis [20,21]. In line with this, we found that urinary MG-H1 levels were associated with systolic and diastolic blood pressure, pulse pressure, mean arterial blood pressure, and total and LDL cholesterol after adjustment for age, BMI or WHR, and HOMA-IR, but not with any glycemic measurements. To the best of our knowledge, no previous studies have reported the relationships between urinary MG-H1 levels and cardiometabolic parameters. Nonetheless, a study that measured total urinary AGE levels showed positive associations with systolic and diastolic blood pressure in healthy individuals and patients with metabolic syndrome [6]. However, methylglyoxal has been positively associated with fasting glucose levels in patients with T2DM on glucose or lipid lowering treatments [22]. The discrepancy may be the result of differences in the study populations, as our participants had normal glucose tolerance and did not use any medications including glucose- or lipid-lowering drugs. Our data suggest that urinary MG-H1 levels may be important to assess the risk of cardiovascular disease, but this should be confirmed by future longitudinal studies. 

The present study has some limitations. We did not obtain data on dietary AGEs and alcohol intake, which may have influenced our findings. In addition, circulating AGE levels were not determined, hence we were unable to show their relationships with urinary AGEs. The study was performed in non-vegetarian, obese Caucasian women with normal glucose tolerance, thus our results may not be generalizable to other populations such as men, vegetarians, non-obese individuals, and patients with hyperglycaemia, as well as other ethnicities. Furthermore, owing to the cross-sectional nature of the study, we cannot establish causality. Despite these limitations, our participants were metabolically well-characterized. We were thus able to adjust our findings for potential confounders, such as age, BMI or WHR, and HOMA-IR. 

In summary, we have demonstrated that urinary CML levels were positively associated with obesity and markers of insulin resistance, while urinary MG-H1 levels were associated with cardiovascular parameters including blood pressure and lipid levels in obese, but otherwise healthy women. Our findings suggest that urinary AGE levels may be useful for assessing future risk of cardiometabolic syndrome in obese individuals; however, future longitudinal studies including both genders and individuals without T2DM are highly warranted.

## Figures and Tables

**Table 1:** Participant characteristics.

Parameters	Mean ± SD
Age, years	39.98 ± 8.72
Weight, kg	90.96 ± 12.40
BMI, kg/m^2^	32.29 ± 4.05
WHR	0.92 ± 0.05
Systolic blood pressure, mmHg	123.28 ± 13.91
Diastolic blood pressure, mmHg	82.26 ± 9.62
Pulse pressure, mmHg	41.03 ± 7.04
Mean arterial pressure, mmHg	95.92 ± 10.71
Total cholesterol, mmol/L	5.04 ± 0.72
HDL, mmol/L	1.37 ± 0.28
LDL, mmol/L	3.10 ± 0.68
Triglycerides, mmol/L	1.09 ± 0.48
Fasting glucose, mmol/L	5.33 ± 0.31
2 h glucose, mmol/L	5.75 ± 1.09
Fasting insulin, µU/L *	0.91 ± 0.31
2 h insulin, µU/L *	1.73 ± 0.89
HOMA-IR, (µU/L) /(mmol/L) *	0.29 ± 0.29
HOMA-B, % *	1.94 ± 0.26
ISI_0, 120_ *	1.85 ± 1.31
Urinary CML, µg/mL *	0.26 ± 0.26
Urinary MG-H1, µg/Ml *	0.66 ± 0.28

* Log transformation was performed. *n* = 56 for fasting insulin, HOMA-IR, HOMA-B, and ISI_0, 120_. BMI, body mass index; WHR, waist-to-hip ratio; HOMA-IR, homeostatic model of insulin resistance; HOMA-B, homeostatic model of insulin secretion; HDL, high-density lipoprotein; LDL, low-density lipoprotein; ISI, insulin sensitivity index; CML, carboxymethyl lysine; MG-H1, methylglyoxal-hydroimidazolone; SD: standard deviations.

**Table 2:** Univariable analyses between advanced glycation end products (AGE) levels and cardiometabolic parameters.

Parameters	Urinary CML Levels, µg/mL		Urinary MG-H1 Levels, µg/mL	
	*r* Value	*p* Value	*r* Value	*p* Value
Age, years	−0.10	0.27	−0.17	0.20
BMI, kg/m^2^	0.29	0.02	0.17	0.19
WHR	0.22	0.08	0.14	0.28
Systolic blood pressure, mmHg	0.19	0.13	0.33	0.01
Diastolic blood pressure, mmHg	0.13	0.32	0.26	0.04
Pulse pressure, mmHg	0.19	0.10	0.27	0.04
Mean arterial pressure, mmHg	0.16	0.24	0.34	0.02
Total cholesterol, mmol/L	0.11	0.40	0.26	0.04
HDL, mmol/L	−0.05	0.70	0.03	0.81
LDL, mmol/L	0.13	0.32	0.22	0.09
Triglycerides, mmol/L	0.07	0.58	0.12	0.35
Fasting glucose, mmol/L	0.14	0.22	-0.07	0.57
2 h glucose, mmol/L	0.23	0.07	0.02	0.83
Fasting insulin, µU/L	0.37	0.004	0.06	0.62
2 h insulin, µU/L	0.36	0.01	0.14	0.29
HOMA-IR, (µU/L) /(mmol/L)	0.37	0.004	0.05	0.66
HOMA-B, %	0.36	0.01	0.09	0.49
ISI_0, 120_	−0.22	0.05	−0.08	0.49

All values were log transformed. Pearson correlations were performed. *n* = 56 for fasting insulin, HOMA-IR, HOMA-B, and ISI_0, 120_. BMI, body mass index; WHR, waist-to-hip ratio; HOMA-IR, homeostatic model of insulin resistance; HOMA-B, homeostatic model of insulin secretion; HDL, high-density lipoprotein; LDL, low-density lipoprotein; CML, carboxymethyl lysine; MG-H1, methylglyoxal-hydroimidazolone.

**Table 3:** Multivariable analyses of urinary AGE levels with cardiometabolic parameters.

Parameters	Urinary CML Levels, µg/mL		Urinary MG-H1 Levels, µg/mL	
	β coefficient (95% CI)	*p* Value	β coefficient (95% CI)	*p* Value
Systolic blood pressure, mmHg	0.03 (−0.01, 0.08)	0.11	0.06 (0.02, 0.11)	0.01
Diastolic blood pressure, mmHg	0.02 (−0.02, 0.07)	0.37	0.05 (0.01, 0.17)	0.01
Pulse pressure, mmHg	0.06 (−0.02, 0.14)	0.14	0.08 (0.01, 0.15)	0.02
Mean arterial pressure, mmHg	0.02 (−0.02, 0.07)	0.22	0.06 (0.01, 0.14)	0.01
Total cholesterol, mmol/L	0.04 (−0.02, 0.11)	0.71	0.07 (0.01, 0.13)	0.01
HDL, mmol/L	0.05 (−0.03, 0.14)	0.33	0.02 (−0.06, 0.16)	0.46
LDL, mmol/L	0.03 (−0.07, 0.14)	0.70	0.09 (−0.001, 0.19)	0.05
Triglycerides, mmol/L	−0.06 (−0.26, 0.13)	0.36	0.04 (−0.13, 0.23)	0.65
Fasting glucose, mmol/L	0.01 (−0.01, 0.01)	0.16	−0.01 (−0.03, 0.01)	0.54
2 h glucose, mmol/l	0.06 (−0.02, 0.15)	0.71	−0.01 (−0.09, 0.07)	0.85
Fasting insulin, µU/L	0.21 (−0.02, 0.44)	0.06	−0.06 (−0.29, 0.16)	0.58
2 h insulin, µU/L	0.25 (−0.01, 0.51)	0.05	0.04 (−0.21, 0.35)	0.72
HOMA-IR, (µU/L) /(mmol/L)	0.22 (−0.02, 0.47)	0.06	−0.06 (−0.31, 0.17)	0.56
HOMA-B, %	0.16 (−0.04, 0.37)	0.12	−0.04 (−0.24, 0.15)	0.66
ISI_0, 120_	−0.04 (−0.16, 0.07)	0.41	0.97 (−0.11, 0.10)	0.97

All values were log transformed. Adjusted β coefficients were reported. *n* = 56 for fasting insulin, HOMA-IR, HOMA-B, and ISI_0, 120_. Regression models for cardiovascular parameters were adjusted for age, body mass index, and HOMA-IR. Regression models for metabolic parameters were adjusted for age and body mass index. CI, confidence interval; HOMA-IR, homeostatic model of insulin resistance; HOMA-B, homeostatic model of insulin secretion; HDL, high-density lipoprotein; LDL, low-density lipoprotein; CML, carboxymethyl lysine; MG-H1, methylglyoxal-hydroimidazolone.
